# Radio frequency surface plasma oscillations: electrical excitation and detection by Ar/Ag(111)

**DOI:** 10.1038/s41598-017-10170-y

**Published:** 2017-08-29

**Authors:** Giulia Serrano, Stefano Tebi, Stefan Wiespointner-Baumgarthuber, Stefan Müllegger, Reinhold Koch

**Affiliations:** Institute of Semiconductor and Solid State Physics, Johannes Kepler University Linz, 4040 Linz, Austria

## Abstract

We electrically excite surface plasma oscillations on a Ag(111) single crystal by alternating electric charging at radio frequency. The radio frequency signal energy of 2.2 *μ*eV, used to induce surface plasma oscillations, is about 5 to 6 orders of magnitude lower than the plasmon energies reachable by optical excitation or electron impact. The detection of the surface plasma oscillations is achieved by nano-fabricated 2D single-crystal sensor-islands of Ar atoms, which are shown by imaging with a scanning tunneling microscope to restructure in response to the radio frequency surface plasma oscillations, providing nanometer spatial resolution and a characteristic decay time of ≈150 ns.

## Introduction

Charge density oscillations in metals are collective plasma oscillation of the itinerant electrons with the ‘plasmon’ as energy quantum^1^. The scientific quest for controlling their interaction with electromagnetic waves has founded the field of plasmonics, leading to new devices for subwavelength optics, sensing and metrologies^2, 3^. Plasma oscillations at the surface of a conducting material exist in different types – distinguishable by their dispersion behavior^4–7^ – and down to very small (practically zero) energies, as predicted by theory^4, 7, 8^. (i) The conventional surface plasmon originates from the existence of the sample surface (metal-dielectric interface) in three-dimensional electron systems^4^. (ii) In two-dimensional (2D) electron systems, purely two-dimensional surface plasmons may exist, as demonstrated, e.g., by Nagao *et al*. on a monoatomic layer of Ag atoms on Si(111)^9^. (iii) In the presence of surface/bulk state interactions, e.g. via dynamic screening, a certain type of 2D plasmon can exist: the so-called acoustic surface plasmon. Its excitation has been realized experimentally by electron energy loss spectroscopy on Be(0001)^10^ and the (111) facets of Cu, Ag, and Au^11–13^, where the partially occupied band of Shockley surface states forms a 2D electron gas. The excitation of conventional surface plasmons was achieved by optical excitation as well^14^. Combining optical excitation with detection by a scanning tunneling microscope (STM) has facilitated the investigation of surface plasmons with (sub)nanometer spatial resolution^15–20^. To date, the smallest energies achieved experimentally for exciting surface plasmons have been about 100 meV^9, 12, 13, 21^.

Here we demonstrate detection of radio-frequency (rf) surface plasma oscillations with energies as small as 2.2 *μ*eV at the surface of a Ag(111) single crystal, utilizing nano-fabricated 2D single-crystal sensor-islands of Ar atoms imaged with atomic resolution by STM. The plasma oscillations are excited electrically by rf alternating electric charging of the sample mounted inside a rf-STM instrument. The sensor-islands, which are physisorbed on Ag(111) by weak van-der-Waals bonds below 30 K^22^, turn out to be sensitive to the rf surface plasma oscillations via a plasma-oscillation-induced enhancement of the atomic surface diffusion, providing nanometer spatial resolution and a characteristic decay time of ≈150 ns.

## Results

The Ag(111) sample is connected to the output of a rf voltage generator in parallel to a dc voltage source. Details of the rf-circuit and electronics have been described elsewhere^23, 24^. Rf biasing leads to an alternating-in-time electric charging, which forces longitudinal charge density oscillations in the skin layer of the Ag sample, i.e. longitudinal surface plasma oscillations^1, 4^, at the generator frequency. We have applied continuous-wave (cw) as well as pulsed rf voltage biasing to the sample, for varying total on-time *t*_on_. To guarantee constant rf voltage amplitude at the sample surface during all our experiments, we set a fixed generator frequency. At an applied frequency of *f* = 530 MHz, which corresponds to an energy of *hf* = 2.2 *μ*eV, the surface plasma oscillations penetrate only the surface skin layer^25, 26^ of the metal sample with a thickness of ≈80 nm at 5 K, (see supplementary material).

### Sensor islands

**Figure 1:**
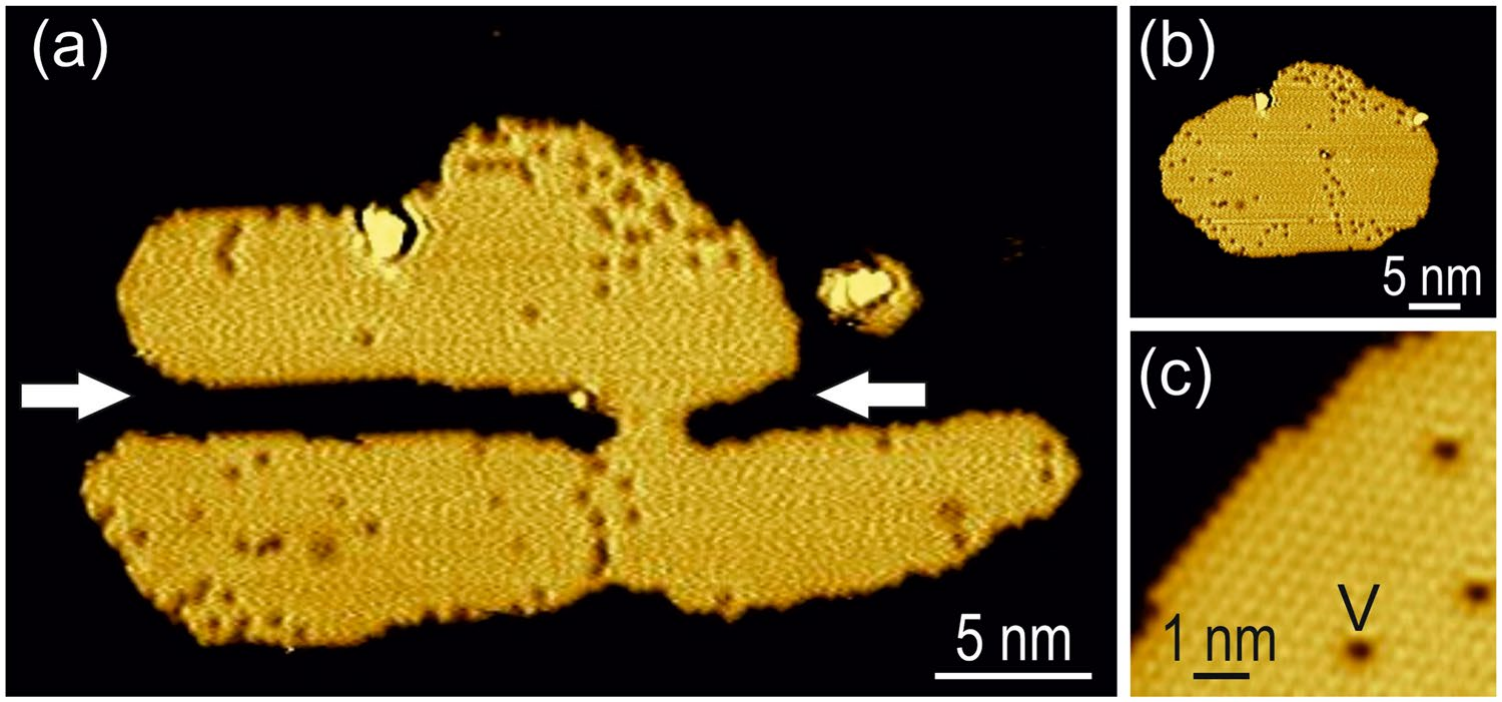
(**a**) Nanometer-sized sensor island: nano-fabricated 2D-island of Ar/Ag(111) imaged by STM at 5 K (+0.4 V, 70 pA, *z*-scale 0.2 nm); arrows mark artificial nano-fabricated channels (see text). (**b**) Same 2D-island as in (**a**) before nano-fabrication. (**c**) Atomic-resolution image of Ar 2D-island; single Ar vacancy is labeled V.

For the experimental detection of the surface plasma oscillations, excited by rf voltage biasing, we utilize nanofabricated 2D single-crystal islands of physisorbed noble-gas atoms imaged with atomic resolution by STM before and after the rf excitation; we denote them as *sensor islands* hereafter. To minimize STM-tip effects, we have carefully maintained a clean metallic tip state and have repeated the experiments with several different tips (after tip-forming on the bare Ag substrate). Figure 1a shows exemplarily a typical nanofabricated sensor island imaged by STM at 5 K. It is based on a 2D-island of Ar on Ag(111) that exhibits a strongly non-equilibrium shape obtained after cutting lateral channels out of the island. The channels are marked by arrows in Fig. 1a and have been created artificially by the controlled removal of Ar atoms with dc-STM manipulation^27^, as shown in the Supplementary Fig. S1. We demonstrate below that these channels are suitable experimental probes for detecting surface plasma oscillations. For comparison, Fig. 1b displays the 2D-island of (a) before the nano-fabrication step, i.e. exhibiting its natural compact equilibrium shape. Figure 1c shows a magnified view revealing the regular hexagonal Ar atomic lattice of the 2D-islands with Ar-Ar distance of 0.39 nm, in agreement with the literature;^22, 28, 29^ a single Ar vacancy is labeled V. The equilibrium-shape islands (Fig. 1b) are stable against continuous dc-STM imaging (+0.4 to +1.3 V and 50–200 pA) for at least 12 h, similar to a full Ar monolayer on Ag(111)^22^. More importantly, also the non-equilibrium sensor islands (Fig. 1a) are longterm stable against dc-STM imaging at 5 K, as shown in the Supplementary Fig. S2.

### Sensor response

**Figure 2:**
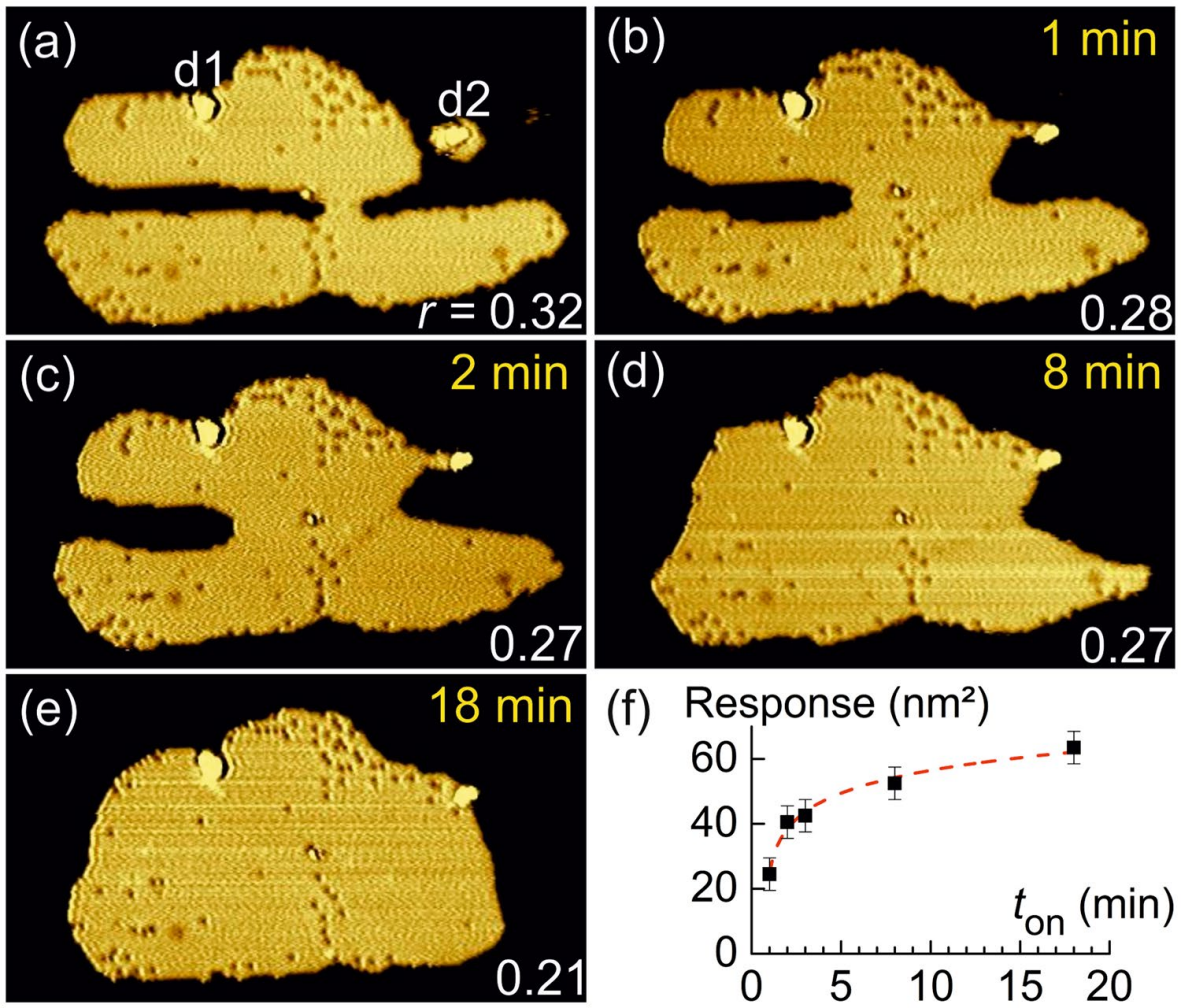
Response of sensor island to cw rf-excitation. (**a**) Sensor island before rf-excitation imaged by STM (53 × 30 nm^2^, +0.4 V, 70 pA). (**b**–**e**) Same sensor island as in (**a**) after successive cw rf-excitation (530 MHz, *P*_thres_ + 4 dB) for accumulating on-time, *t*_on_, as labeled; *r* is the perimeter-to-area ratio in nm^−1^; the total projected area of the island is 730 nm^2^. (**f**) Dependence of the sensor response (see text) on the duration of cw-rf-excitation *t*_on_; dashed line: numerical fit by logarithmic function $a+b\cdot \,\mathrm{ln}({t}_{{\rm{on}}}+c)$ with fit parameters *a* = 36 ± 3, *b* = −9 ± 1 and *c* = −0.7 ± 0.2.

The sensor islands respond to the rf excitation (ac biasing of substrate) with characteristic structural changes. Figure 2 shows representative examples for cw-excitation at 530 MHz; pulsed excitation causes similar changes but to a weaker extent (see below). Cw-excitation for a duration of *t*_on_ = 1 min causes a restructuring of the sensor island that leads to a gradual closing of the channels, clearly revealed by comparing the images (a) before and (b) after cw-excitation. In the following, we denote the restructuring as ‘sensor response’ and quantify it further below. We observe that sensor response neither adds nor subtracts a significant amount of argon atoms to/from each sensor island, leaving their total areas unaffected (as quantified below). The sensor response against dc tunneling is zero. Comparing Fig. 2a–e, notice the defects, labeled d1 and d2, are unaffected by the rf excitation and the large island merging with the small island close to d2. Obviously, sensor response proceeds via a directed displacement of Ar atoms across the Ag surface by additional elementary diffusion, which tends to minimize the surface free energy of the island by decreasing its perimeter-to-area ratio. The additional diffusion is absent (frozen) at dc-voltage tunneling conditions at 5 K, where only edge-diffusion of Ar atoms is observed, i.e. diffusion along the same atomic row starting off from a kink site. Notice that edge diffusion alone cannot explain sensor response, because the closing of the channels requires the formation of Ar ledge atoms in the Ar island, i. e. Ar atoms moving out of an edge row forming a new edge one row in front of the old one. Additional cw-excitation of the sensor island for accumulating *t*_on_ leads to a further closing of the channels (Fig. 2c,d). Apparently, closure of the channels is achieved after a total accumulated *t*_on_ of about 8 min (Fig. 2d), and after *t*_on_ = 18 min the sensor island finally adopts a compact equilibrium-like shape similar to the shape of original Ar 2D-islands prior to nanofabrication (compare Fig. 2e with 1b).

We have quantified the sensor response by determining the total (projected) area of laterally displaced Ar atoms in the STM image of a sensor island after rf excitation (Fig. 2b–e) compared to the undisturbed sensor island prior to rf excitation (Fig. 2a). Figure 2f shows that, starting from zero, sensor response increases monotonically with increasing duration of cw-excitation, *t*_on_, and finally saturates. Simultaneously, the perimeter-to-area ratio of the sensor islands decreases monotonically with accumulating *t*_on_ (within the experimental error). The characteristic saturation behavior of the sensor response has been observed for all studied sensor islands (more than 50) and it is consistent with minimizing the island’s surface free energy at long rf exposures. These experimental findings are crucial, because they evidence the directed, i.e. non-random, nature of the underlying physical process.

Within the experimental rf-power range of our method, we have observed a nearly linear dependence of sensor response on the rf power (at fixed frequency), as shown in the Supplementary Fig. S3 for 530 MHz. The respective curve exhibits a lower threshold of *P*_thres_≈3 dBm generator output power. This corresponds to an rf-voltage amplitude at the sample in the millivolt range, considering the damping of the rf circuitry^24^. The value of *P*_thres_ has been found to be frequency dependent; the above value is the smallest threshold observed within our investigated frequency range of 200–3000 MHz and applies to a range of approximately 530 ± 120 MHz. Up to our available maximum bandwidth of 2 GHz, no experimental indications for a resonant frequency of sensor response have been observed.

### Independence of the electric field between sample and tip

**Figure 3:**
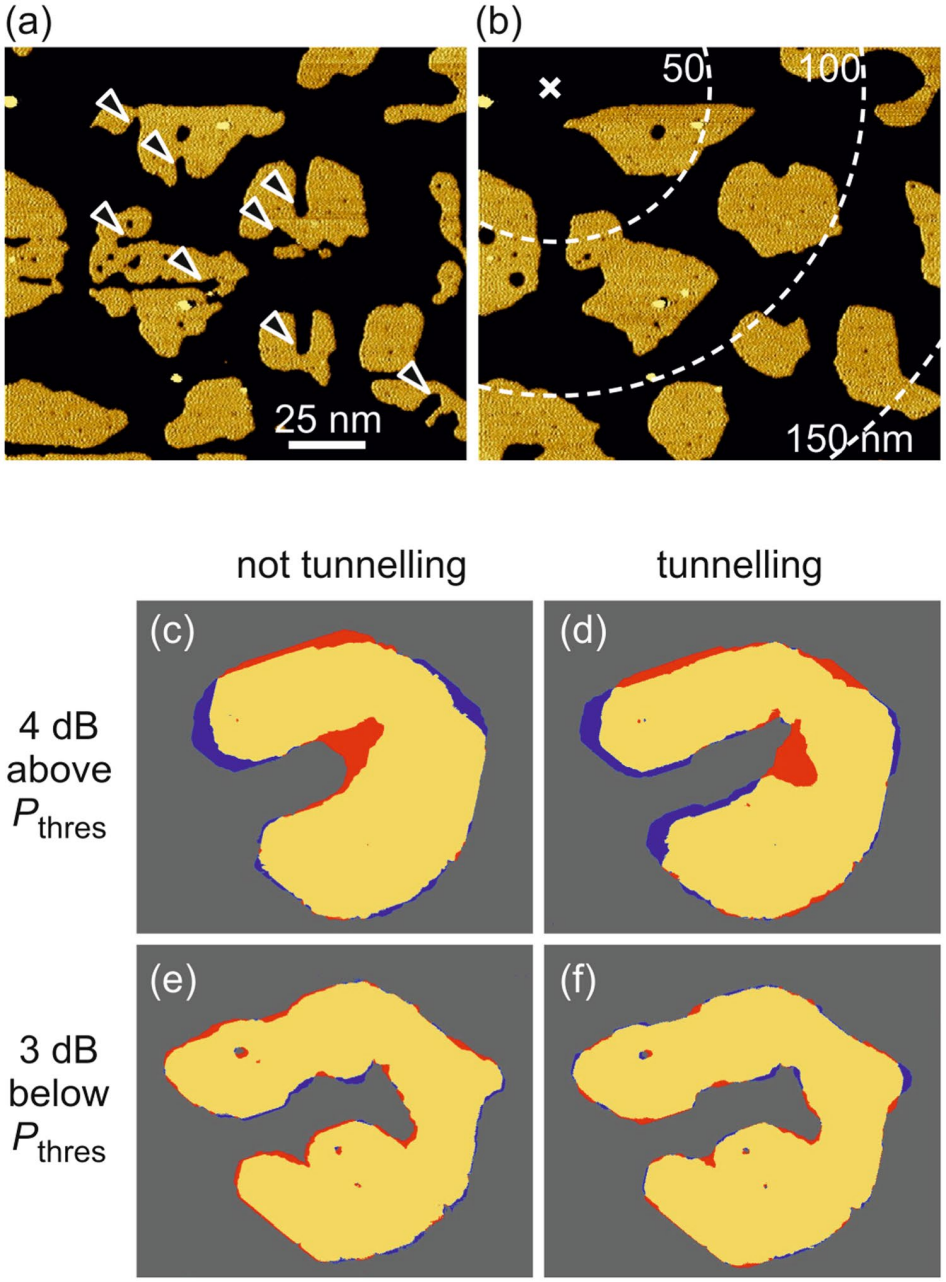
Sensor response is independent of STM tip position. (**a**,**b**) Sensors, marked by arrows, before (**a**) and after (**b**) cw-excitation (530 MHz, *t*_on_ = 5 min); cross: tunnel position of STM tip ( + 0.4 V) during excitation; dashed lines: radial distances from tip position. (**c**–**f**) Difference images of sensor response, obtained by subtracting STM images before and after rf-excitation at different rf-power and tunnel conditions as labeled; yellow, red, and blue colors mark zero change, accumulation, and removal of Ar atoms.

Figure 3a shows sensor islands with artificially fabricated channels in both horizontal and vertical direction, marked by arrows. After cw rf-excitation for 5 min all sensors have strongly responded (Fig. 3b), although during the excitation the STM tip was placed over the pristine substrate several tens of nanometers away from the sensors (tip position marked by cross). Repeating this experiment with the STM tip laterally displaced to various different positions across the whole image frame yields the same result. This finding corroborates that sensor response occurs independent of the lateral position of the STM tip during rf excitation. Apparently, sensor response is based on a mechanism that is independent of the close-up range of the tip apex and isotropic in the surface plane. This observation is consistent with the surface propagation of surface plasma oscillations^4^ that are strongly localized to the surface and propagate parallel to the surface over very large distances. On Ag(111), which is the lowest-loss photonic metal, the lateral propagation length of surface plasmons is ≈9 *μ*m in the infrared regime^17^ and exceeds 100 *μ*m at optical frequencies^25^. In our case of 530 MHz, we have confirmed sensor response up to a surface area of 400 × 400 nm^2^ on Ag(111) by manual piezo control (only limited by the scan range of our STM instrument at 5 K).

To study the effect of the tunneling current and the electric field between sample and STM tip on sensor response, we have performed rf-biasing experiments with different tip-sample separations (*z*) and rf power levels. Figure 3c–f juxtapose the respective results. For better clarity, sensor response is displayed as difference images with red (blue) color marking sensor area, where Ar atoms have been accumulated (removed) by rf-excitation. In a first step, we retract the STM tip perpendicularly away from the sample by 200 nm, thus dramatically suppressing electron tunneling and, furthermore, reducing the electric field between STM tip apex and sample by a factor of about 200. Nevertheless, even at such extreme non-tunneling conditions, sensor response is clearly evidenced by the observed channel closing after 5 min of cw rf-biasing with rf power of 4 dB above *P*_thres_ (Fig. 3c). The response is practically indistinguishable from that at tunneling conditions (Fig. 3d). Obviously, sensor response is independent of tunneling electrons. Moreover, it is not affected by the change of the magnitude of the electric field between tip and sample, thus ruling out a ‘simple’ effect of the electric field between sample and STM tip. The latter is corroborated by repeating the experiments at a decreased power level of 3 dB below *P*_thres_ resulting in zero response at both non-tunneling (Fig. 3e) and tunneling conditions (Fig. 3f). The respective decrease of power corresponds to a factor of 1/2 concerning the rf electric field amplitude, compared to a factor of 1/200 for tip retraction. The observed result is indeed crucial: There is no response in Fig. 3f, although the electric field between tip and sample is at least 100 times larger compared to the retracted case with sufficient rf power (Fig. 3c). Obviously, sensor response is uncorrelated with the strength of the dc- and rf-electric fields between sample and STM tip (i.e. for *z* > 1 nm). Notice that our argumentation holds independent of the precise value of the rf-voltage amplitude at the tunneling junction, which depends on damping in the rf circuit.

### Temporal response of Ar sensor islands

**Figure 4:**
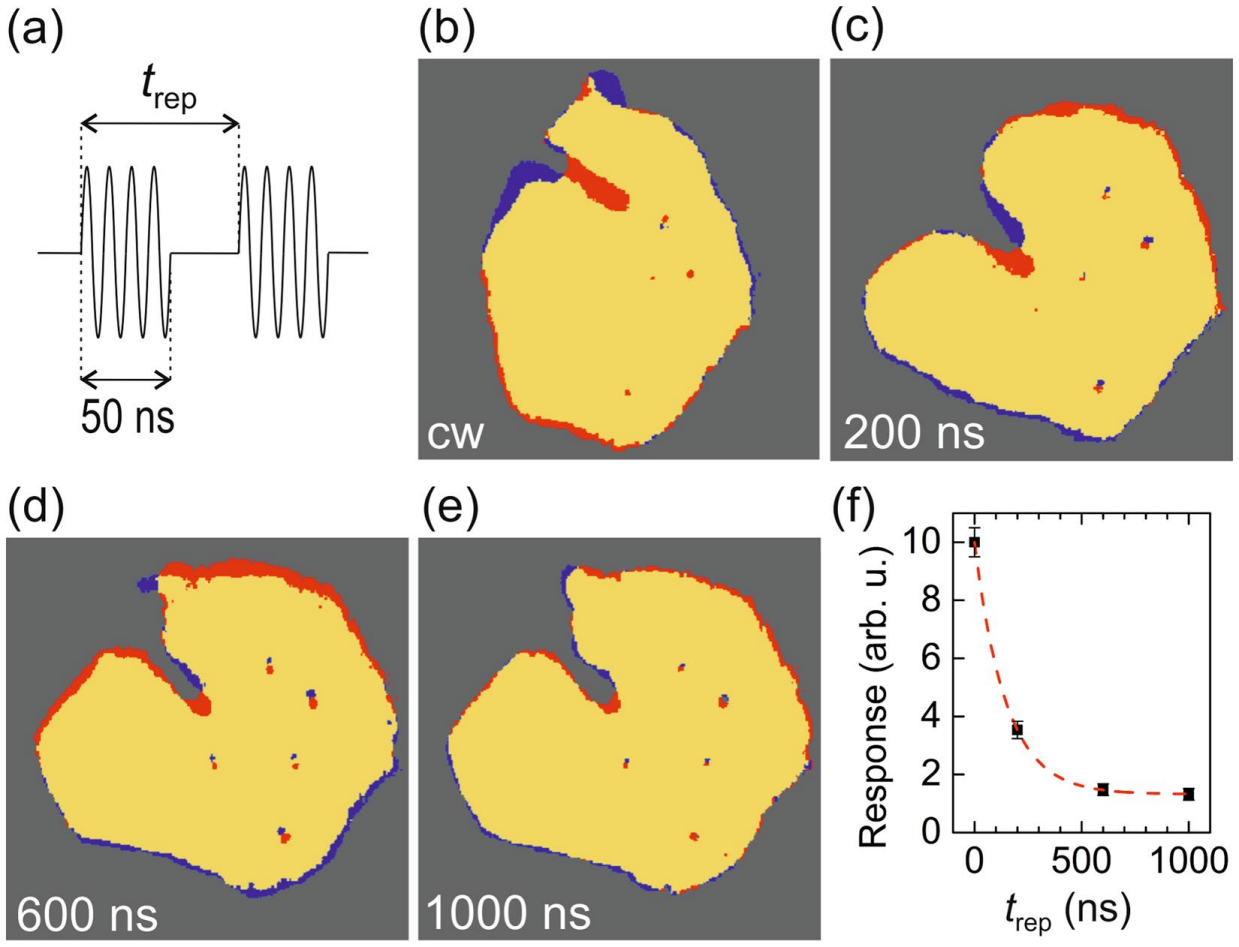
Sensor response to pulsed rf-biasing (530 MHz, *P*_thres_ + 7 dB) at non-tunneling conditions. (**a**) Schematics of periodic rf pulses. (**b**–**e**) Difference images; yellow, red, and blue colors mark zero change, accumulation, and removal of Ar atoms; (**b**) after 1 min cw-excitation; (**c**–**e**) after excitation by 1.2 × 10^9^ periodic 50 ns-pulses (equivalent of 1 min cw) with repetition time *t*_rep_ = 200, 600, and 1000 ns. (**f**) Dependence of response on *t*_rep_; dashed line: numerical fit $\propto \exp ({t}_{{\rm{rep}}}/\tau )$.

To gain insight into the temporal response of the sensor islands, we have investigated sensor response to pulsed rf-biasing. For all of our pulsed experiments, we have obtained practically the same results for tunneling as well as non-tunneling conditions; for brevity we discuss herein only the results at non-tunneling conditions. We have applied pulse trains (Fig. 4a) consisting of periodic 50 ns-pulses of frequency 530 MHz with different values of repetition time *t*_rep_; each pulse train contained the same total number of 1.2 × 10^9^ pulses, equivalent of a total on-time of 1 min of the rf-biasing. Although having the same total on-time of 1 min, pulsed excitation with *t*_rep_ = 200 ns yields a more than 50% smaller response than cw-excitation (compare Fig. 4b,c). Moreover, increasing the pulse period to 1000 ns further decreases the response (Fig. 4d,e). Our pulsed experiments indicate that the sensor response depends on the duty cycle of rf-biasing, i.e. the fraction of pulse duration (here *t*_puls_ = 50 ns) to repetition time (*t*_rep_). Note, however, that the total applied rf-power is the same for all of our pulse experiments. Our results therefore indicate that the individual perturbations of the Ar lattice induced by single 50-ns-pulses cannot explain the sensor response, because the process of creating new Ar atomic rows (see section sensor response) by a single pulse becomes reversed effectively for an increasing delay time, *t*_rep_−*t*_puls_, until the next pulse. Accordingly, the (collective) dynamic process underlying the sensor response exhibits a time constant *τ* in the range of the chosen *t*_rep_ value. Figure 4f shows a quantitative evaluation of the *t*_rep_-dependence of the response. Numerical fitting an exponential function yields a value of *τ* = 147 ± 1 ns for the time constant of sensor response. Similar values of decay times have been reported recently for the collective mechanical vibrations of weakly physisorbed molecules on Au(111)^30^. The decay times of excited (surface) plasmons and (surface) phonons on Ag(111), however, are typically about six orders of magnitude shorter^31, 32^.

### Thermal effects

According to Berthold *et al*.^33^, a monoatomic layer of Ar desorbs from a Ag(111) surface at temperatures above 35 K. Restructuring of isolated Ar 2D islands by thermally induced diffusion may occur already at lower temperatures (see supplementary material). Therefore, we have to carefully exclude rf-induced heating as alternative explanation of the observed sensor response. The Ag(111) sample is directly mounted to and therefore in very good thermal contact with the STM head of our ‘beetle’-type Createc LT-STM. Throughout the experiments the sample temperature has been permanently controlled via a Si-diode temperature sensor mounted to the STM head in very good thermal contact to the sample. By external heating of the STM head (including the sample), we have studied the effect of temperature on sensor response. We observe that heating at 10 K for 60 min without applying rf-biasing causes a sensor response of similar magnitude as rf-biasing at 5 K for 5 min. Note that sensor response at 5 K and dc tunneling conditions is zero (see section sensor response and supplementary material). Thus, explaining the observed sensor response by thermal effects requires heating of the sample to temperatures well above 10 K, which is not the case in our experiments: (i) The observed independence of the sensor response on the flow of tunnel current (Fig. 4) rules out local Joule heating at the tunnel junction as possible origin. Joule heating relies on electric current flowing^26^, whereas sensor response occurs even at zero tunnel current. (ii) The observed independence of the sensor response on the electric field between sample and STM tip rules out heating by microwave radiation. Sensor response occurs in the near-field of the sample surface, where the rf field is known to exchange only reactive power with the sample, i.e. not to dissipate electric power. (iii) We can also rule out a significant temperature increase of the sample due to Joule heating by the rf current that is responsible for the alternating charging of the sample. With a spectrum analyzer (Agilent N9020A) connected in series with the rf transmission line^30^ we obtain a root-mean-square (rms) value of *I*_rf_≈400 *μ*A for an rf power of 4 dB above *P*_thres_. If the total electric power, ${I}_{\mathrm{rf},\mathrm{rms}}^{2}\cdot R$, was dissipated exclusively in the skin layer of the sample (with resistance *R*) during *t*_on_ = 5 min of cw rf-biasing, this would lead to a temperature increase of the Ag sample of ${\rm{\Delta }}T={I}_{{\rm{rms}}}^{2}\cdot R\cdot {t}_{{\rm{on}}}/{c}_{{\rm{p}}} < 2\cdot {10}^{-5}$ K (see supplementary material), neglecting heat transfer from the sample to the cold STM head. This tiny temperature increase cannot explain the sensor response. (iv) If dielectric loss in the transmission line or sample mounting is significant, we expect to measure a respective temperature increase by the Si-diode temperature sensor. In comparison to (iii), we have measured with the Si-diode a slight warming of the sample of <0.2 K upon 5 min of cw rf-biasing with a power of 4 dB above *P*_thres_, which we attribute to dielectric loss. Since the sample is in very good thermal contact with the Si-diode temperature sensor, this finding confirms that the only measurable heating is too small to explain the sensor response. (v) The observed independence of sensor response on the tunnel distance rules out thermovoltage effects. Thermovoltage acts as a constant voltage source in series with the sample bias voltage, causing an exponential dependence on the tunnel distance^16^. Summarizing our above findings, thermal effects cannot explain the observed sensor response.

## Discussion

The main findings obtained with the Ar sensor-islands are: (1) Rf-biasing at 530 ± 120 MHz of the Ag(111) sample causes a restructuring of the sensor islands (sensor response). The restructuring is (2) independent of the presence of the STM-tip and (3) independent of dc and ac electric fields between sample and STM tip. (4) Restructuring induced by periodically pulsed rf-biasing depends on the delay time between subsequent pulses, *t*_rep_−*t*_puls_ (while keeping the overall rf on-time fixed). (5) Thermal effects cannot explain the restructuring.

It is known that ac biasing, in general, electrically excites longitudinal electron density oscillations, i.e. (non-radiative) longitudinal plasma oscillations^1, 4^. They are accompanied by a periodic-in-time transfer of momentum from the driving electric field to the conduction electrons, causing their alternating acceleration. Electrical excitation of plasma oscillations is possible at continuous frequency-values irrespective of their wave vector *k*. This freedom in *k* is a huge advantage over optical plasmon excitation; for the latter, simultaneous matching of frequency and *k* is essential^4^. The absence of a resonance behavior observed for sensor response (see above) is consistent with the freedom in *k* of electrical excitation.

Longitudinal rf plasma oscillations periodically-in-time add (remove) conduction electrons to (from) the skin layer of the Ag sample analogous to alternating charging of a capacitor by an ac voltage. In the present case, the skin layer of the Ag sample forms one capacitor electrode and the STM housing that surrounds the sample forms the counter-electrode. We estimate a respective total capacitance of about 0.75 pF based on the geometrical dimensions of our sample and STM head. At an rf power level of 4 dB above *P*_thres_, the electric potential of the Ag sample is periodically-in-time changed by ≈20 mV root-mean-square, thus polarizing the physisorbed Ar atoms. Accordingly, the electrostatic energy of the Ar atoms periodically alternates by about ±20 meV. This value lies slightly above the formation energy of Ar ledge atoms of ≈13 meV (see supplementary information). Notice, that the value of *P*_thres_ observed in our experiments corresponds to a periodic change of the sample’s electric potential of about ±13 mV root-mean-square. Therefore, by stimulating charge fluctuations and thus disturbing the Van der Waals bonding of the Ar atoms of the sensor islands, the rf-induced surface plasma oscillations may indeed cause the observed sensor response.

On the time scale of several nanoseconds and more, studied herein, the surface plasma oscillations (whose oscillation period lies in the range of nanoseconds, as well) couple the dynamics of the conduction electrons and the dynamics of the atomic lattice in the skin layer of the sample. For plasmons at optical frequencies, two mechanisms are known to occur simultaneously: (i) The decay of surface plasmons can generate (surface) phonon-polaritons^5, 10^; (ii) due to the surface-confined electric field of the plasmon, electrostriction can strain the surface atomic lattice mechanically^34, 35^. In the present case of surface plasma oscillations at radio frequency, we consider (direct) phonon excitation by alternating rf charging of the sample as unlikely, because the wavelength of the plasma oscillation is expected to be orders of magnitude larger than the phonon wavelength: extrapolation of the phonon dispersion of Ag(111)^36^, which is nearly linear at small energies, yields a phonon wavelength of ≈270 *μ*m for an energy of 2.2 *μ*eV. In comparison, the wavelength of rf surface plasma oscillations at the same energy is ≈57 cm, i. e. considerably larger than the sample dimensions of ≈1 cm, indicating a coherent alternating charging of the entire Ag surface. Moreover, phonon propagation, which occurs at the scale of the sound velocity, is approximately 10^5^ times slower than the rf-induced charge dynamics; thus, phonons are affected only by the time-average of the alternating electric charging, which is constant in time. These considerations support that rf-biasing does not resonantly excite phonons in the Ag sample.

The electrostrictive response of the atomic lattice occurs at a time-scale of pico-seconds^37^. The excitation of surface plasma oscillations by rf-biasing of the substrate is expected to readily induce periodic-in-time electrostrictive displacement of the Ag atoms in the skin layer of Ag(111) at a frequency similar to the rf (here 530 MHz). The amplitude, Δ*z*, of the respective vertical electrostrictive component contributes exponentially to the tunnel current via the well-known relation ${I}_{{\rm{tunnel}}}\propto {e}^{-{\rm{\Delta }}z}$. We estimate Δ*z* ≤ 5 pm based on the change of the dc tunnel current upon switching on/off the rf biasing. Although detectable, mere vertical displacements are unlikely to be the origin of the observed sensor response.

Based on our experimental results and the above considerations, we interpret the observed sensor response and related atomic displacements, evidenced by Figs 2–4, by an enhancement of the diffusion of Ar atoms that is induced by the (low-energy) radio frequency surface plasma oscillations. Most likely, a decrease of the diffusion barrier is caused by the electric ‘near’-field of the forced rf longitudinal surface plasma oscillation, affecting the dispersive (van der Waals-type) forces of the physisorbed Ar atoms. Due to the dynamic nature of this field, the sample experiences electric field components, both, perpendicular as well as parallel to the sample surface. A similar effect of an external rf electric field on weak dispersive bonding has been reported for biomolecules^38^.

## Conclusion

We have demonstrated that (i) alternating electric charging at radio frequency of a Ag(111) substrate generates longitudinal surface plasma oscillations with energies as small as a few *μ*eV and (ii) Ar islands are suitable analytical probes for detecting them. In addition to forced plasma oscillations, two different types of low-energy surface plasmons (eigenmodes) have been reported to exist on Ag(111), see introduction, with their dispersion relation monotonically approaching *k* = 0^8, 11^. We therefore cannot exclude the possibility of resonant electrical excitation of surface plasmons (eigenmodes) by rf-biasing, whenever the frequency of the external rf-biasing coincides with the plasmonic eigenfrequency. As shown for plasmonic nanoparticles, in such a case the wavelength of the surface plasmon may indeed be 1–2 orders of magnitude larger than the size of the sample^39^. Our study suggests electrical excitation of surface plasmons as promising new technique, which lifts the experimental challenges of optical excitation of matching frequency an *k*-vector simultaneously. Our sensor islands will provide a sensitive tool for future analytical studies on surface plasma oscillations at very small energies, i.e. 5–6 orders of magnitude below the energies reachable by electron loss spectroscopy or optical excitation.

## Methods

Experiments were performed in ultrahigh vacuum (<10^−10^ mbar) with a rf-adapted Createc low-temperature scanning tunneling microscope (STM) operated at 5 K^23^. A tungsten tip, electrochemically etched and thermally deoxidized above 1070 K, acts as imaging probe as well as movable ground-electrode against the Ag(111) single-crystal sample, prepared by repeated cycles of Ar^+^ ion sputtering (600 eV) and thermal annealing at 720 K. When mounted inside the LT-STM, the Ag(111) sample is surrounded by the gold-plated-copper body of the STM head, forming a capacitor with circa 0.75 pF. Notice, that the capacitance of the tunnel junction is much smaller and thus negligible here; typical values of the geometric and the quantum capacitance of the tunnel junction are 10^−18^ to 10^−15^ F and 10^−18^ F, respectively^40–43^. The spacing to the STM head (vacuum) is at least 3 mm on each side of the sample. After cooling the sample to 5 K, the STM chamber was flooded for 1 min with Ar gas at a pressure of $5\cdot {10}^{-7}$ mbar, yielding Ar islands on top of the bare Ag(111) surface with a constant height of one monoatomic Ar layer (densely-packed), a compact shape, and a diameter ranging from about 30 to 100 nm; the nominal Ar coverage of the Ag surface is ≈0.3 monolayers. For the rf experiments, the sample is simultaneously biased by two independent voltage sources, i.e. a dc-voltage source (Createc LT-STM sample bias) and a rf-voltage source (Rohde & Schwarz SMA100A low-noise signal generator), connected in parallel with the help of a bias-tee (Mini circuits ZFBT-4R2GW + ). Cavity effects of the sample mounted inside the STM head and impedance matching are, both, not crucial for the observed sensor response: In our case, the lowest cavity resonance modes lie above 820 MHz, i. e. well above our applied frequency of 530 MHz. The frequency dependent damping of our transmission line may only affect the value of the power-threshold for observing sensor response, but cannot explain the sensor response (see main text).

## Electronic supplementary material

Supplementary Information
