# Supplementary material for: Radio frequency surface plasma oscillations: electrical excitation and detection by Ar/Ag(111)
Source: Sci Rep. 2017 Aug 29;7:9708. doi: 10.1038/s41598-017-10170-y (PMC5574974; doi:10.1038/s41598-017-10170-y)
Supplement: Supplementary file 1 — Supplementary Information [file 41598_2017_10170_MOESM1_ESM.pdf]

SUPPLEMENTARY MATERIAL

Radio frequency surface plasma oscillations: electrical excitation and detection by Ar/Ag(111)

Giulia Serrano, Stefano Tebi, Stefan Wiespointner-Baumgarthuber, Stefan Müllegger,* and Reinhold Koch
Institute of Semiconductor and Solid State Physics,
Johannes Kepler University Linz, 4040 Linz, Austria.

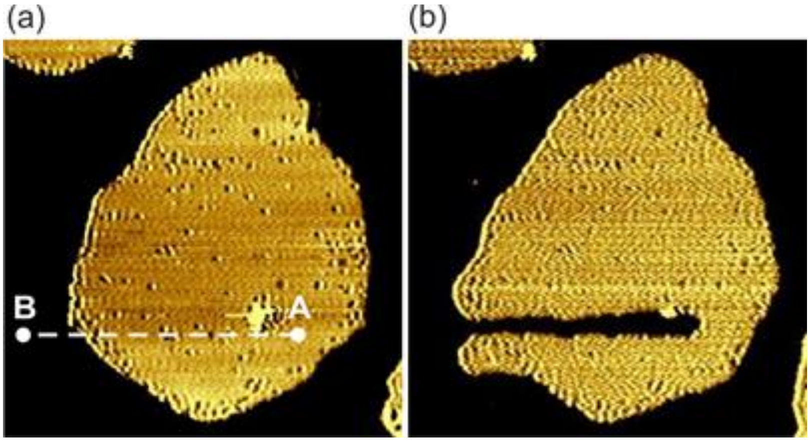

Figure S1. Fabrication of non-equilibrium Ar islands. STM images (68x68 nm^2, +0.4V, 50pA) of an Ar island before (a) and after (b) STM manipulation. During the Ar island manipulation the tip is moved along the dashed line from a position A to B by applying +0.01V and 5nA.

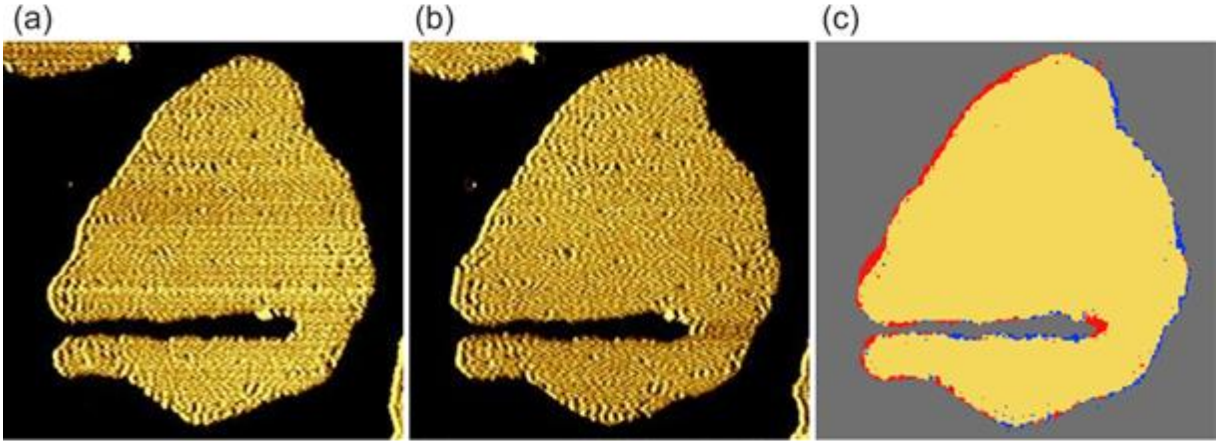

Figure S2. Stability of nanostructured non-equilibrium Ar 2D-islands upon continuous dc-STM imaging. Panels (a) and (b) show two STM images (68x68 nm^2, +0.4 V, 50 pA) of the same island before and after 13 h of continuous dc-STM imaging. (c) "difference image" obtained by subtracting the STM images (b) minus (a), "after minus before"; image subtraction was done pixel-by-pixel. The area of the Ar 2D-island where no changes have occurred is displayed in yellow color; red (blue) color marks that area of the island, were Ar atoms have been accumulated (removed) during the total time of continuous STM imaging.

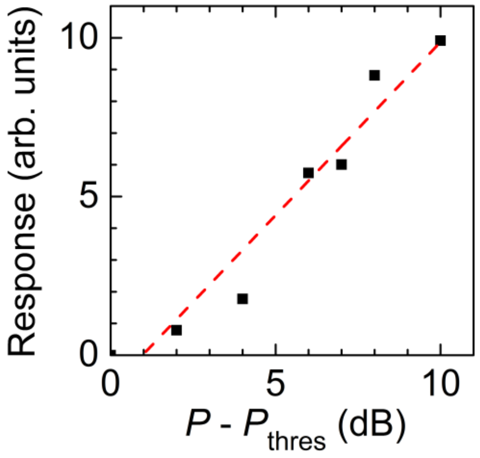

Figure S3. Power dependence of the response of the sensor islands; dashed line: numerical fit by linear function. The observed threshold power P_{thres} of approximately 3 dBm (generator output power) corresponds to a zero-to-peak amplitude of the rf voltage at the sample of a few mV, due to the damping of the rf circuitry line.

Skin layer thickness of Ag(111) at 5K

The thickness of the skin layer is given [1] by $\delta = \sqrt{\rho/(\mu\pi f)}$. Ag has an electrical resistivity of $\rho = 1.3 \cdot 10^{-11}$ Ωm [2] at 5 K and a magnetic permeability of $\mu \approx \mu_0 = 4\pi \cdot 10^{-7}$ Vs/Am. For a frequency of $f = 530$ MHz, the skin thickness is $\delta \approx 80$ nm.

Ar on Ag(111): physisorption and thermal desorption

The use of Ar for the detection of mechanical motion relies on the well-suited strength of the Ar bonds for this new type of experiments. On the one hand, the Ar-Ag bond is strong enough to suppress desorption at 5 K. Temperature-programmed-desorption experiments of Ar/Ag(111) revealed a zero-order-like desorption process for the Ar monolayer starting close to 35 K ($k_B T \approx 3$ meV) [3] corresponding to a desorption energy of 97 meV per atom. This is in good agreement with the value of 99 meV per atom reported for the latent heat of condensation of Ar/Ag(111)[4]. In general, physisorbates (such as rare gases like Ar) on noble metals exhibit adsorption energies of the order of ≈40 meV per atom [5]; for instance, a value of 56 meV per atom was reported for Xe/Pt [6]. On the other hand, the Ar-Ar bond is strong enough to prevent Ar 2D-islands from dissociating when rf-voltage is applied. The cohesive energy of a 3D Ar crystal is 78 meV per atom, resulting in a melting temperature of 84 K [7].To date, no experimental value for the surface diffusion energy barrier of Ar on Ag(111) has been reported in the literature to the best of our knowledge.

Formation energy of Ar ledge atoms in Ar islands on Ag(111):

We estimate the formation energy of Ar ledge atoms, as follows: We have observed (see main text) the absence of sensor response at 5 K ($k_B T \approx 0.4$ meV) and the onset of sensor response at about 10 K. Since sensor response relies on the formation of Ar ledge atoms (see main text), we estimate that the formation energy of Ar ledge atoms in Ar islands on Ag(111) is of the order of 1-10 meV. Based on the literature value of 78 meV [7] for the cohesive energy of fcc

argon (where each Ar atom has 12 nearest neighbors), the Ar-Ar dimer binding energy is estimated to 78/12 = 6.5 meV. The formation of an Ar ledge atoms in the 2D island requires the breaking of two Ar-Ar dimer bonds in total, i.e. an energy of 13 meV.

Nano-fabrication of motion sensors (non –equilibrium 2D islands)

Non-equilibrium Ar 2D-island ("motion sensors") are fabricated by lateral manipulation with the STM tip, which selectively displaces Ar atoms as shown in Figure S1. The STM tip is placed over the island (position A, Figure S1,a) and moved closer to the surface by lowering the bias voltage to +0.01 V and raising the tunneling current to 1-8 nA. In a next step, the STM tip is moved horizontally along a straight path, indicated by dashed line, towards the final position (B) that lies outside of the island. At position B, the STM tip is retracted away from the sample by restoring the "normal" tunneling conditions used for STM imaging (+0.4 V, 50 pA). The procedure is repeated up to ten times until the final channel has been formed, see Figure S1,b.

Longterm stability of nano-structured non-equilibrium Ar 2D-islands against continuous dc-STM imaging and storage at 5 K

Non-equilibrium 2D-islands are stable upon dc-STM imaging at +0.4 V and 50–100 pA. Figure S2 shows the same island before (a) and after (b) 13 h of continuous dc-STM imaging at +0.4 V and 50 pA. Panel (c) shows a "difference image" obtained by a pixel-to-pixel subtraction of the STM images (b) minus (a). The difference image helps to visualize structural changes of the island during the total time of STM imaging. The area of the Ar 2D-island where no changes have occurred is displayed in yellow color; red (blue) color marks that area of the island, where Ar atoms have been accumulated (removed) during the total time of continuous STM imaging; a grey background color was chosen for better clarity. Obviously, continuous dc-STM imaging does not significantly affect the shape of the sensor island; in particular, we find no sensor response (closing of the channel, see main text). The only structural change visible in figure S2,c is the diffusion of Ar atoms along the outermost atomic row of the rim of the sensor island, which we denote as "edge diffusion" in the main text. Notice that edge diffusion is observed at the outermost Ar atomic row of the sensor 2D-islands in all our experiments independent of rf excitation.

Heating of the sample by the rf current

We estimate the temperature increase of the Ag sample, ΔT, caused by Joule heating of the rf current, I_{rf} at a frequency of 530 MHz. The latter flows in the skin layer of the Ag sample. We measure a root-mean-square (rms) value of $I_{\mathrm{rf,rms}} \approx 400$ µA. This value of the (displacement) current is consistent with the experimental value of the damping of the rf transmission line of about -28 dB from generator to junction, measured independently. During the on-time of the cw rf-biasing, $t_{\mathrm{on}} = 300$ s, a total electric energy ${I_{\mathrm{rf,rms}}}^2 \cdot R \cdot t_{\mathrm{on}}$ is dissipated as heat, Q, in the skin layer of the sample, where $R = \varrho_{\mathrm{Ag,5K}} \cdot L/A$ is the electrical resistance of the skin layer. The resistance is obtained from the specific resistivity of Ag at 5 K of $\varrho_{\mathrm{Ag,5K}} = 1 \cdot 10^{-11}$ to $5 \cdot 10^{-11}$ Ωm [2,8], the length $L = 5.8$ mm of the current path, and the cross-sectional area, $A = 9 \cdot 10^{-10}$ m^2, of the skin layer; the values of L and A follow from the geometrical dimensions of our Ag sample and the skin layer thickness of 80 nm (see above). The total dissipated heat, Q, increases the temperature of the whole Ag sample, which has a mass of $m = 0.94$ g and a specific heat of $c_p = 0.2253$ J/(kg K) at 5 K [8]. Accordingly, we calculate for the respective temperature increase, $\Delta T = Q/(m \cdot c_p)$, of the Ag sample a value of $\Delta T \approx 1.8 \cdot 10^{-5}$ K.

References:

[1] J. D. Jackson, Classical Electrodynamics, 4th ed. (John Wiley & Sons, Inc., New York, Chichester, Weinheim, Brisbane, Singapore, Toronto., 1999).
[2] R. G. Chambers, Proc. R. Soc. Lond. A 215, 481 (1952).
[3] A. Damm, K. Schubert, J. Gudde, and U. Höfer, Phys. Rev. B 80, 205425 (2009).
[4] A. Unguris, L. W. Bruch, E. R. Moog, and M. B. Webb, Surf. Sci. 109, 522 (1981).
[5] R. Gomer, Rep. Prog. Phys. 53, 917 (1990).
[6] D. L. Meixner and S. M. George, J. Chem. Phys. 98, 9115 (1993).
[7] N. Bernardes, Phys. Rev. 112, 1534 (1958).
[8] D. R. Smith and F. R. Fickett, J. Res. Natl. Inst. Stand. Technol. 100, 119-171 (1995).
